# Synergistic influence of phosphorylation and metal ions on tau oligomer formation and coaggregation with α-synuclein at the single molecule level

**DOI:** 10.1186/1750-1326-7-35

**Published:** 2012-07-23

**Authors:** Georg Nübling, Benedikt Bader, Johannes Levin, Jenna Hildebrandt, Hans Kretzschmar, Armin Giese

**Affiliations:** 1 Center for Neuropathology and Prion Research, Ludwig-Maximilians-Universität, Feodor-Lynen-Str. 23, 81377, Munich, Germany; 2 Neurologische Klinik und Poliklinik, Klinikum der Universität München, Marchioninistr. 15, 81377, Munich, Germany

**Keywords:** α-Synuclein, Metal ion, Oligomer, Phosphorylation, Tau, Iron, Aluminium, GSK-3 beta, Alzheimer’s disease, Parkinson’s disease

## Abstract

**Background:**

Fibrillar amyloid-like deposits and co-deposits of tau and α-synuclein are found in several common neurodegenerative diseases. Recent evidence indicates that small oligomers are the most relevant toxic aggregate species. While tau fibril formation is well-characterized, factors influencing tau oligomerization and molecular interactions of tau and α-synuclein are not well understood.

**Results:**

We used a novel approach applying confocal single-particle fluorescence to investigate the influence of tau phosphorylation and metal ions on tau oligomer formation and its coaggregation with α-synuclein at the level of individual oligomers. We show that Al^3+^ at physiologically relevant concentrations and tau phosphorylation by GSK-3β exert synergistic effects on the formation of a distinct SDS-resistant tau oligomer species even at nanomolar protein concentration. Moreover, tau phosphorylation and Al^3+^ as well as Fe^3+^ enhanced both formation of mixed oligomers and recruitment of α-synuclein in pre-formed tau oligomers.

**Conclusions:**

Our findings provide a new perspective on interactions of tau phosphorylation, metal ions, and the formation of potentially toxic oligomer species, and elucidate molecular crosstalks between different aggregation pathways involved in neurodegeneration.

## Background

Several neurodegenerative diseases comprise neuronal or glial deposits consisting mainly of protein tau, such as Alzheimer’s neurofibrillary tangles (NFTs) or Pick bodies, and are therefore termed “tauopathies“. In Alzheimer’s Disease (AD), tau exhibits pathological hyperphosphorylation [1,2], allowing both histological diagnosis by use of tau antibodies against disease specific phosphorylation sites [3,4] and, to a certain extent, even in vivo diagnosis by determination of the protein’s phosphorylation status in cerebrospinal fluid [5,6].

One of the most important tau kinases is Glycogen Synthase Kinase 3β (GSK-3β), which has been shown to create AD specific phospho sites on tau in vitro [7], in cell culture [8,9] and in vivo [10,11]. Some [12,13] but not all [14] authors reported increased GSK levels in AD brains. GSK-3β is colocalized with NFTs [15], and the distribution of its active form in AD brains coincides with the appearance of tau pathology [16].

Tau phosphorylation by GSK-3β promotes the formation of paired helical filaments (PHF) in vitro [17-19], though data concerning the relevance of this effect vary [20]. An enhancing impact of GSK-3β on tau aggregation was also demonstrated in cell culture and in vivo [21-23], supporting a possible role of this kinase in AD pathogenesis. Furthermore, phosphorylation influences metal ion induced tau aggregation. Several studies demonstrated that tau phosphorylation enhances Al^3+^ induced aggregation [24,25] or even is a prerequisite for such aggregation [26,27].

The influence of aluminium on tau aggregation has been extensively studied, since the metal ion was shown to induce NFT-like deposits in mammalian brain after intracerebral injection [28]. Though aluminium levels were found to be raised in AD hippocampus [29] and the metal ion was colocalized with NFTs and early tau deposits in brain sections [30,31], its relevance to AD pathogenesis is still unclear, especially due to the inconsistent outcome of epidemiological studies [32].

In vitro studies examining effects of ferric iron (Fe^3+^) yielded results resembling those obtained for aluminium. Fe^3+^ also induces the aggregation of phosphorylated protein tau [27], is colocalized with NFTs [30,33,34] and elevated in AD hippocampus and amygdala [35]. Furthermore, Fe^3+^ induces α-synuclein (α-syn) aggregation [36-38].

Co-deposits of tau and α-syn have been found in several neurodegenerative diseases, and interactions between these two proteins recently gained increasing interest. α-Syn has been detected in NFTs of AD, progressive supranuclear palsy (PSP) and corticobasal degeneration (CBD) [39], whereas tau was located in Lewy bodies of patients with Dementia with Lewy bodies (DLB) [40]. In vitro, tau in solution requires inducers like heparin for filament formation, whereas the protein readily polymerizes in presence of α-syn without inducers [41].

Furthermore, the minimal α-syn concentration necessary for fibril formation is reduced in presence of tau, and some of the fibrils formed in presence of both proteins comprise tau and α-syn segments [41]. Considering that both proteins are located in the cytoplasmic compartment of neurons, and that minimal concentrations of α-syn oligomers can cross-seed tau aggregation [42], interactions of tau and α-syn may be relevant for pathological protein aggregation in neurodegenerative diseases.

While established methods of monitoring tau and α-syn aggregation like Thioflavin T assay or atomic force microscopy yield important insights in fibril formation and the formation of large oligomers, they are not suitable to directly monitor single protein interactions or interactions of different proteins. It was demonstrated that small oligomer species are on-pathway to tau filament formation [43]. Furthermore, it is increasingly recognised that prefibrillar small oligomers rather than the large NFTs might be responsible for neuronal and synaptic loss [44-46]. To investigate the influence of phosphorylation on tau oliomer formation and interactions between tau and α-syn, we employed fluorescence correlation spectroscopy (FCS) and scanning for intensely fluorescent targets (SIFT) to investigate the influence of phosphorylation and trivalent metal ions on tau aggregation and its coaggregation with α-syn. These methods allow monitoring of oligomerization processes at the single molecule level even at nanomolar protein concentrations [47,48]. Moreover, the possibility to label proteins with different dyes allows the investigation of tau and α-syn interactions at the level of individual oligomers.

## Results

### Tau phosphorylation verified by western blot and SIFT

**Figure 1:**
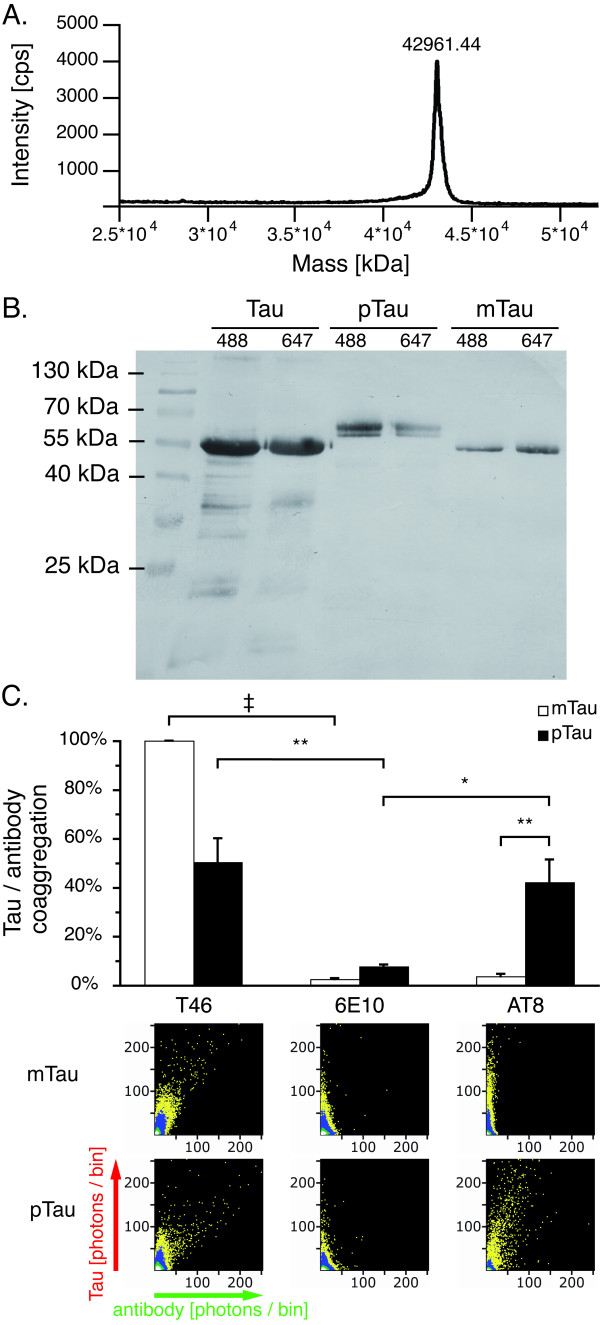
** Tau phosphorylation verified by western blot and SIFT analysis.A.** Mass spectroscopy showed that recombinant human tau (isoform 5, 42967 Da) is of high purity. **B.** While mock phosphorylation (mTau) does not influence SDS-PAGE mobility of recombinant tau, a typical band shift is observed upon tau phosphorylation (pTau). **C.** SIFT analysis showed labeling of pTau oligomers with the phosphorylated tau specific AT-8 antibody in presence of 1% DMSO indicating antibody binding, while no coaggregation was observed with mTau. Data was normalized against the coaggregation level of mTau with the T46 antibody, which does not require tau phosphorylation. 2D histograms depicting antibody (x-axis) and protein (y-axis) interactions show coaggregates of T46 with both pTau and mTau, while AT-8 only coaggregates with pTau. A scheme describing the appearance of mixed aggregates in 2D histograms is included in Figure 3. Upon combining AT-8 and mTau, only DMSO induced tau aggregates are visible along the y-axis, similar to the control antibody 6E10. Measurements were taken from 12 independent samples; each sample was measured four times. Levels of significance are displayed as * = p < 0.05; ** = p < 0.01; ‡ = p < 0.001.

Mass spectroscopy showed that recombinant protein tau is of high purity and does not contain significant amounts of cleavage products (Figure 1A). Notably, fluorescence labeling of tau with Alexa dyes did not alter the protein’s electrophoretic mobility, though mass spectroscopy showed an increase in tau’s molecular weight after labeling (data not shown). We confirmed in vitro phosphorylation of human recombinant tau by demonstrating a typical band shift of the protein in western blot (see Figure 1B) [17]. We further evaluated whether the SIFT method can be employed to detect phosphorylated protein tau by labeling with Alexa-488 tagged antibodies AT-8 and T46. While T46 detects both phosphorylated and unphosphorylated tau, the AT-8 antibody requires the protein to be phosphorylated at specific sites (Goedert et al, 1995). The Aβ specific antibody 6E10 was used as a negative control. SIFT analysis shows that both T46^488^ and AT-8^488^, but not 6E10^488^ bind to phosphorylated tau^647^ (pTau, Figure 1C), while only T46^488^ binds to mock phosphorylated tau^647^ (mTau, also see materials and methods section). 

### Influence of phosphorylation on tau aggregation induced by aluminium and DMSO

**Figure 2:**
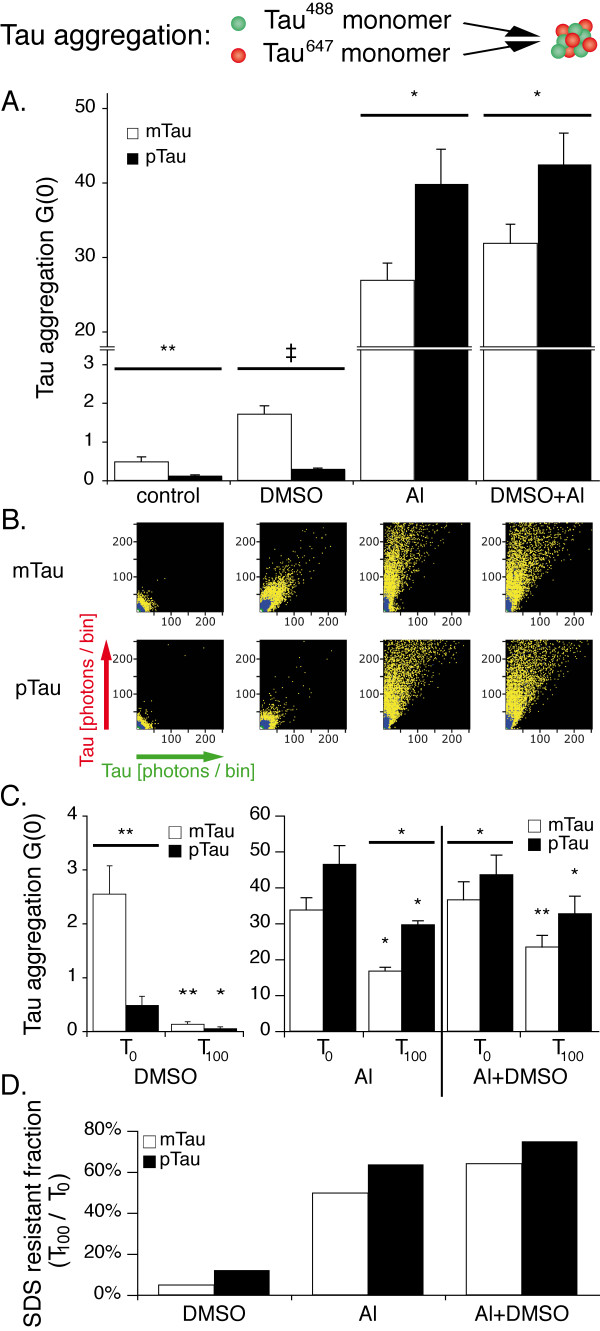
** Al**^**3+**^**promotes the formation of SDS stable tau oligomers more efficiently after tau phosphorylation.A.** Cross-correlation amplitudes (G (0)) show mild proaggregatory activity of the organic solvent DMSO, which was significantly higher for mTau. Al^3+^ promotes intense protein oligomerization. Measurements were taken from 15 independent samples; each sample was measured four times. **B.** 2D histograms of detected photons indicate the formation of smaller oligomers in presence of DMSO (mean 76 monomers per oligomer for mTau, 26 monomers for pTau) compared to Al^3+^ (mean 220 to 240 monomers per oligomer). **C.** Cross correlation amplitude immediately before (T_0_) and 100 min after (T_100_) addition of SDS to the aggregation assay. Significant reductions of aggregation levels at T_100_ are indicated by the symbols over the error bars, while significant differences between pTau and mTau are indicated by the symbols over the wide bars. Measurements were taken from 7 independent samples. **D.** Cross correlation amplitude at T_100_ normalized against T_0_ shows increased SDS-resistance of Al^3+^ induced oligomers compared to DMSO. Levels of significance are displayed as * = p < 0.05; ** = p < 0.01; ‡ = p < 0.001.

Our previous studies revealed that the organic solvent DMSO and the metal ion aluminium (Al^3+^) induce the formation of distinct tau oligomer species [49]. In order to evaluate the influence of protein phosphorylation on the effects of these aggregation inducers, we employed GSK-3β to create phosphorylated tau (pTau) and compared its aggregation behavior to mock phosphorylated tau (mTau). Our findings show that oligomerization of both pTau and mTau can be induced by 1% DMSO, with mTau showing a higher rate of aggregation than pTau (p < 0.001, Figure 2A). Conversely, in presence of 10 μM Al^3+^, pTau oligomerization exceeds mTau (p < 0.05), with the overall aggregation level being distinctly higher compared to DMSO for both pTau and mTau (also see Additional files 1 and 2). 

2D histograms of detected aggregates show the formation of distinct oligomer species in presence of DMSO and Al^3+^ (Figure 2B). In presence of DMSO, tau protein shows only moderate aggregation, while Al^3+^ enhances the formation of larger oligomers with higher fluorescence intensity.

Fluorescence intensity distribution analysis (FIDA) of tau oligomers induced by DMSO yielded larger aggregates for mTau (mean 76 molecules) than for pTau (mean 26 molecules). In presence of Al^3+^, large tau oligomers comprising on average 240 (pTau) and 220 (mTau) molecules occurred within 15 minutes, while the overall aggregation level was higher for pTau (Figure 2A, for aggregation dynamics also see Additional file 3). The combination of DMSO and Al^3+^ increased aggregation levels compared to Al^3+^ alone predominantly for mTau (also see Additional files 1 and 2).

Furthermore, we examined the SDS stability of oligomers generated by the different enhancers (Figure 2C, D). While DMSO induced aggregates dissolve upon addition of the ionic detergent SDS in a final concentration of 0.2%, more than 50% of the Al^3+^ induced oligomer signal remains stable 100 min after addition of SDS.

In conclusion, Al^3+^ promotes rapid formation of detergent resistant oligomers, preferably of phosphorylated protein tau. Conversely, DMSO induced aggregation is diminished upon tau phosphorylation.

### Influence of tau phosphorylation on the coaggregation of monomeric tau and α-synuclein in presence of metal ions and organic solvents

Since the role of tau and α-syn coaggregation is increasingly recognized, we investigated the effect of tau^488^ phosphorylation on its coaggregation with α-syn^647^. As previous work has shown a major influence of Fe^3+^ on α-syn oligomerization [36-38], we also used Fe^3+^ at a final concentration of 10 μM in our assay.

**Figure 3:**
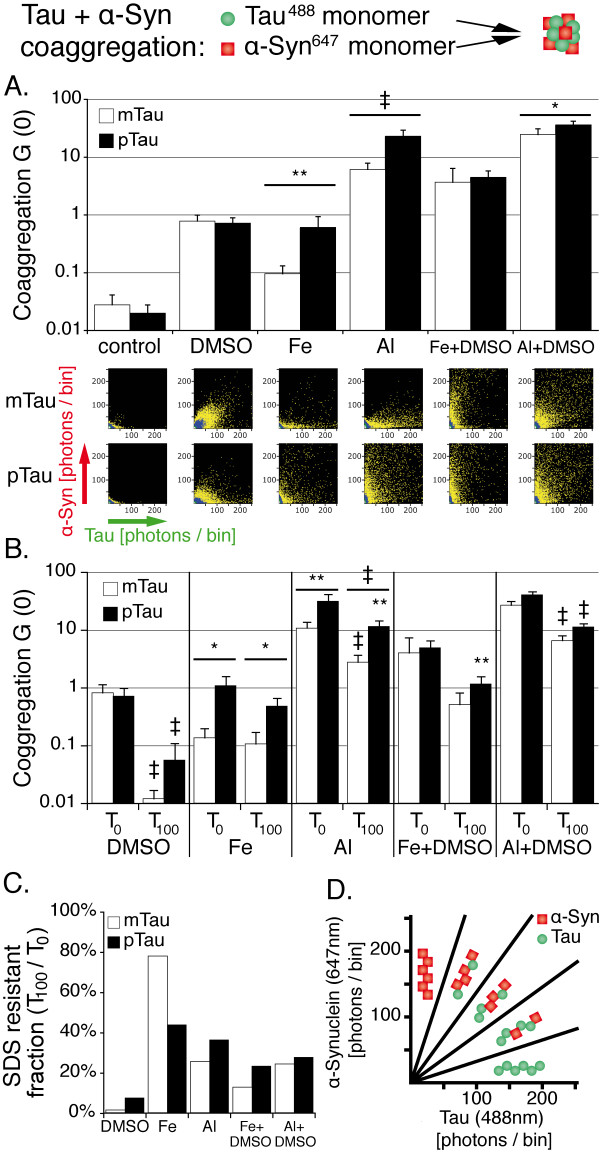
** Al**^**3+**^**promotes stronger formation of SDS stable tau and α-syn coaggregates than Fe**^**3+**^**, preferably after tau phosphorylation.A.** Cross-correlation amplitudes (G (0)) show mild proaggregatory activity of DMSO and Fe^3+^, while Al^3+^ promotes intense protein oligomerization. Combinations of DMSO and metal ions show a synergistic effect especially for mTau. 2D histograms show the formation of smaller oligomers induced by DMSO compared to Al^3+^. **B.** Cross-correlation amplitude before (T_0_) and 100 min after (T_100_) addition of SDS to the aggregation assay. Significant reductions of aggregation levels at T_100_ are indicated by the symbols over the error bars, while significant differences between pTau and mTau are indicated by the symbols over the wide bars. **C.** Cross correlation amplitude at T_100_ normalized against T_0_ shows increased SDS-resistance of metal ion induced oligomers compared to DMSO. **D.** Scheme illustrating the appearance of tau and α-syn aggregates and coaggregates of the two proteins. Higher fluorescence intensities of detected oligomers indicate increased oligomer size. Measurements were taken from 16 independent samples; each sample was measured four times. Levels of significance are displayed as * = p < 0.05; ** = p < 0.01; ‡ = p < 0.001.

In these experiments, 1% DMSO promoted the coaggregation of tau and α-syn in a similar manner for pTau and mTau (Figure 3A). On average, coaggregates were composed of 11 pTau + 8 α-syn monomers or 15 mTau + 12 α-syn monomers, respectively, as determined by FIDA analysis. Fe^3+^ also had a promotional influence on coaggregation, which was higher for pTau (p < 0.01, on average 53 pTau + 10 α-syn and 39 mTau + 3 α-syn monomers per oligomer). Upon combining DMSO and Fe^3+^, an additional effect exceeding the influence of one inducer alone can be seen (also see Additional files 4 and 5), while no significant difference in aggregation levels of pTau and mTau was detectable. Similar to our tau oligomerization experiments, Al^3+^ was the strongest inducer of tau and α-syn coaggregation, with pTau showing higher coaggregation activity than mTau (p < 0.001, Figure 3A; on average 131 pTau + 115 α-syn and 137 mTau + 80 α-syn monomers per oligomer). This difference was still detectable when combining Al^3+^ with DMSO, though to a lesser extent (p < 0.05). Mixed oligomers comprising both tau and α-syn appear scattered around the bisectrix of these histograms, while homogeneous aggregates are located close to the axes, as illustrated in Figure 3D.

Concerning oligomer stability, 0.2% SDS reduced the number of coaggregates formed in presence of every tested agent (Figure 3B, C). Again, coaggregates formed in presence of metal ions proved to be more resistant to SDS than those induced by DMSO, though to an overall lesser extent than the homogeneous tau aggregates. Oligomer sizes before and after addition of SDS as determined by FIDA analysis are provided in Additional file 6. The presence of Al^3+^-induced, SDS stable mixed oligomers was further validated applying gel filtration and fluorescence spectroscopy. In these experiments, Al^3+^ induced oligomers were separated from monomers by gel filtration. SDS stable mixed tau and α-syn oligomers eluted prior to monomers, as demonstrated by cross-correlation and SIFT-2D analysis (see Additional file 7).

Thus, our data show increased tau and α-syn coaggregation after tau phosphorylation in presence of trivalent metal ions, and that the heterogenic oligomers generated by Fe^3+^ and Al^3+^ are more stable to SDS treatment than those formed by DMSO.

### Coaggregation of tau oligomers and monomeric α-synuclein

**Figure 4:**
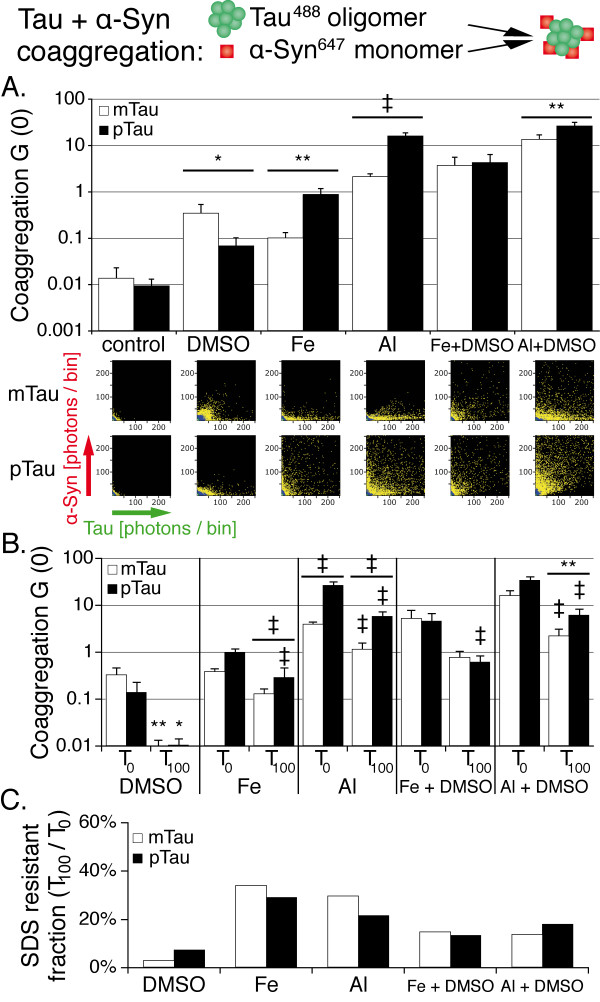
** Monomeric α-syn preferably coaggregates with pTau oligomers in presence of metal ions.A.** Cross-correlation amplitudes (G (0)) show mild coaggregation of α-syn monomers with pre-formed tau oligomers induced by DMSO and Fe^3+^, while Al^3+^ promotes intense coaggregation. Combinations of DMSO and metal ions show a synergistic effect especially for mTau. 2D histograms show the formation of smaller mixed oligomers induced by DMSO compared to the metal ions. **B.** Cross-correlation amplitude before (T_0_) and 100 min after (T_100_) addition of SDS to the aggregation assay. Significant reductions of aggregation levels at T_100_ are indicated by the symbols over the error bars, while significant differences between pTau and mTau are indicated by the symbols over the wide bars. **C.** Cross correlation amplitude at T_100_ normalized against T_0_ shows increased SDS-resistance of metal ion induced oligomers compared to DMSO. Measurements were taken from 20 independent samples; each sample was measured four times. Levels of significance are displayed as * = p < 0.05; ** = p < 0.01; ‡ = p < 0.001.

Giasson et al. showed that tau and α-syn induce each other’s filament formation, and that some of the fibrils formed in presence of both proteins comprise tau and α-syn segments [41]. Such findings suggest that these segments are either assembled by end to end annealing of short filament fragments, or that such fragments act as seeds for both proteins. To evaluate the effect of tau phosphorylation on the interaction of tau oligomers with monomeric α-syn, tau^488^ was first incubated in presence of 1% DMSO, 10 μM Fe^3+^, 10 μM Al^3+^, and combinations of these agents for 90 minutes to generate pre-formed tau oligomers. α-Syn^647^ monomers were then added to the assay and measurements were conducted (Figure 4A). 

Contrary to the experiments with tau monomers, DMSO had a stronger effect on the coaggregation of pre-formed mTau oligomers with monomeric α-syn compared to pTau oligomers (p < 0.05, Figure 4A). Under these conditions, coaggregates contained on average 20 pTau + 2 α-syn monomers or 27 mTau + 5 α-syn monomers, respectively.

pTau oligomer coaggregation with monomeric α-syn exceeded mTau seven- to ninefold in presence of Fe^3+^ (p < 0.01; on average 197 pTau + 14 α-syn or 175 mTau + 3 α-syn monomers per oligomer) and Al^3+^ (p < 0.001; on average 151 pTau + 73 α-syn or 154 mTau + 35 α-syn monomers per oligomer). This difference was markedly reduced upon combining Fe^3+^ and 1% DMSO, which again yielded a synergistic effect compared to the two inducers alone (also see Additional files 8 and 9). The combination of Al^3+^ and 1% DMSO also reduced the difference between pTau and mTau to a two-fold excess of pTau coaggregation (p < 0.01).

SDS (0.2%) reduced the number of oligomers formed in presence of every tested inducer (Figure 4B). Especially aggregates induced by metal ions alone showed enhanced resistance to SDS treatment compared to those formed in presence of DMSO (Figure 4C).

## Discussion

Several studies have dealt with the influence of protein phosphorylation and the metal ion aluminium (Al^3+^) on tau filament formation so far [17-27]. However, the pathophysiological relevance of these factors is still unclear. Whereas most studies were mainly focused on tau filament formation, we applied single molecule fluorescence techniques to investigate the influence of these factors on early tau aggregation steps and oligomer formation. We demonstrate that tau phosphorylation modulates oligomer formation in presence of Al^3+^, and that Al^3+^ induces the formation of distinct large, SDS resistant tau oligomers.

We further demonstrate that coaggregation of tau and α-syn can be observed at the single molecule level, is differentially modulated by tau phosphorylation, and induced by trivalent metal ions. Such coaggregation might be of pathophysiological relevance, since co-deposits of tau and α-syn have been detected in various neurodegenerative diseases [39,40].

### Influence of tau phosphorylation on electrophoretic mobility and antibody interaction

It has been demonstrated that in vitro phosphorylation of human protein tau by GSK-3β can be verified using SDS-PAGE by demonstrating a complete band shift of phosphorylated tau compared to the unphosphorylated protein [17]. We verified efficient tau phosphorylation indicated by a complete band shift compared to the unphosporylated protein (Figure 1B). In addition, we investigated whether fluorescently labeled antibodies can be employed in the SIFT method to verify tau phosphorylation at nanomolar protein concentrations, allowing for possible diagnostic implementations of this method in the future. Our data corroborate that tau phosphorylation was successful and that antibodies can be employed in the SIFT method to identify proteins and posttranslational modifications such as phosphorylation at nanomolar protein concentrations (Figure 1C). Such an assay may provide a valuable tool for future diagnostic applications e.g. the detection of novel specific CSF biomarkers of neurodegeneration.

### Differential influence of phosphorylation on tau aggregation

In vitro studies have yielded conflicting results regarding the influence of tau phosphorylation on its assembly to filaments. To date, both inhibitory and promotional effects of GSK-3β mediated phosphorylation on tau filament assembly were found in absence or presence of polyanionic aggregation inducers [17-20,50,51]. Notably, for Al^3+^ induced tau aggregation, phosphorylation was consistently found to be enhancing or, in some experiments, even a prerequisite [24-27].

However, the methods applied in these studies, such as electron microscopy, thioflavine fluorescence, laser light scattering, SDS-PAGE and western blot, are of limited use in directly examining single molecule interactions at the oligomer level. Furthermore, these techniques often require high concentrations of both protein and aggregation inducer that might not depict physiological conditions, especially given the fact that tau protein readily polymerizes at high concentrations without any inducer [52,53]. In addition, the majority of studies investigating brain Al^3+^ concentrations did not detect concentrations exceeding 10 μg/g brain mass (dry weight) in AD brains [54], which approximately corresponds to 60 μmol/kg (wet weight), while many studies examining the influence of Al^3+^ on tau aggregation in vitro applied Al^3+^ concentrations in the milimolar range [25,27,55].

In this study, we applied confocal single molecule fluorescent techniques to investigate the influence of protein phosphorylation by GSK-3β on tau oligomer formation at the single molecule level at nanomolar concentrations. Exposure to 10 μM Al^3+^ induced the rapid formation of large tau oligomers, while DMSO at a final concentration of 1% led to the formation of smaller oligomers. The large Al^3+^ triggered oligomers contained an average of 220 to 240 molecules as shown by FIDA analysis, and were resistant to treatment with 0.2% SDS, which in contrast readily dissolved the smaller oligomers formed in presence of DMSO. The oligomer sizes observed here were comparable with data presented earlier [49]. Interestingly, phosphorylation by GSK-3β yielded an increase in Al^3+^ induced tau oligomer formation, while oligomer size was comparable for pTau and mTau.

Thus, our data identify tau phosphorylation and physiological concentrations of the metal ion Al^3+^ as synergistic inducers of the formation of SDS resistant tau oligomers even at nanomolar protein concentrations. These findings substantiate the hypothesis that Al^3+^ may play a role in the formation of neurotoxic oligomers even at early stages of neurodegeneration.

### Metal ions and organic solvents enhance tau and α-synunclein coaggregation depending on tau’s phosphorylation status

Co-deposits of tau and α-syn have been found in several neurodegenerative diseases, including AD, PSP, CBD and DLB [39,40]. Since interactions of different proteins are difficult to monitor in vitro, only few studies investigating the interaction of tau and α-syn have been published so far. It was demonstrated that in mixed micromolar solutions of α-syn and tau, both proteins’ minimal concentrations for filament assembly are decreased [41,56]. Moreover, Giasson et al. detected filaments that comprised both tau and α-syn, proving that the two proteins directly interact in mixed solutions [41]. A more recent study showed that α-syn fibrils can be taken up by tau overexpressing cells and induce the formation of phosphorylated triton-insoluble tau oligomers [57]. In vivo studies further demonstrated that tau deposits can be found in mice overexpressing pathologic human α-syn mutations [58].

In this study, we demonstrate that coaggregation of tau and α-syn takes place even at nanomolar protein concentrations, is strongly induced by trivalent metal ions, and differentially modulated by tau’s phosphorylation status. In contrast to the findings of Giasson et al., we did not detect tau and α-syn coaggregation in the absence of aggregation inducers. This apparent discrepancy is most likely explained by the differences in protein concentrations (up to 1000-fold) and the shorter observation time [41]. However, our data demonstrate that tau and α-syn coaggregation occurs even at nanomolar protein concentrations in presence of aggregation inducers. As in our tau aggregation assay, Al^3+^ had the most pronounced effect on coaggregation of α-syn with tau monomers and oligomers. Al^3+^ induces rapid coaggregation of tau and α-syn (see Additional file 3). Fe^3+^ and DMSO also induce coaggregation of the two proteins, though to an overall lesser extent. In our experiments, tau phosphorylation by GSK-3β strongly enhanced the formation of mixed oligomers induced by Al^3+^ or Fe^3+^. Moreover, the mixed oligomers resulting from metal ion induced coaggregation proved to be more resistant to SDS treatment than those formed in presence of DMSO.

We further provide data from FIDA analysis and gel filtration experiments demonstrating the presence and size of mixed tau and a-syn oligomers (see Additional files 6 and 7). Earlier studies applying atomic force micoscropy and SIFT established FIDA-based quantification of oligomer sizes as a reliable method [38].

These results extend the current model of tau and α-syn interaction at early stages of neurodegeneration. As the cytoplasmic concentration of unbound tau and α-syn is low in pre-clinical neurodegeneration, the presence of pro-aggregatory factors may be crucial in the generation of early mixed oligomers. We identify trivalent metal ions and tau phosphorylation by GSK-3β as potential inducers of such oligomer formation even at nanomolar protein concentrations. A time-dependent increase of oligomer concentrations may then induce self-propagating and cross-seeding mechanisms as demonstrated by Giasson and Waxman [41,57].

## Conclusions

In summary, we demonstrate that tau phosphorylation and trivalent metal ions such as Al^3+^ act together in the formation of distinct SDS resistant tau oligomers. Moreover, applying confocal single molecule fluorescence techniques, we show that, under certain conditions, interactions of tau and α-syn can take place even at nanomolar protein concentrations, and result in the formation of mixed oligomers. Notably, the formation of SDS resistant mixed aggregates was induced by physiologically relevant concentrations of trivalent metal ions, and strongly enhanced by tau phosphorylation. Taking into account that a considerable amount of soluble tau exists in a phosphorylated state in neurodegenerative diseases [1,2,59], these findings support a crucial role of specific metal ions such as Al^3+^ and Fe^3+^ in early cytoplasmic aggregation and coaggregation events.

Moreover, our results indicate common pathophysiologic mechanisms of both tau aggregation and cross-seeding phenomena, and might explain the coincidence of tau and α-syn in neuronal deposits. The mixed aggregates described here may provide an interlink of different pathological pathways leading to neurodegeneration, and may serve as promising therapeutic targets for future drug development. Furthermore, we introduce a novel technique of monitoring post-translational modifications at very low protein concentrations that may provide a powerful diagnostic tool in the future.

## Methods

### Expression of human proteins tau and α-synuclein

#### Protein tau

Tau isoform 5 was expressed in E.coli as described previously [49]. In brief, thermocompetent E.coli DE3 (RIL) were transformed, and tau was purified by sterile filtration, cation exchange chromatography and ammonium acid salt precipitation. The transformation vector was a kind gift of Manuela Neumann (ZNP Munich/Zurich).

#### α-Synuclein

α-Synuclein was produced according to established protocols [48]. A stock solution containing 1 mg/ml α-synuclein was prepared in 50 mM tris buffer, pH 7.0.

### Protein labeling

Proteins were labeled with fluorescent dyes Alexa-488-O-succinimidylester and Alexa-647-O-succinimidylester, respectively, as described previously [48]. Tau isoform 5 was incubated with a four-fold molar excess of Alexa-488 or Alexa-647 in 100 mM NaHCO_3_ for 24 h at room temperature. For α-syn, a two-fold molar excess of dye was used. Antibodies T46 (Invitrogen, Carlsbad, CA), AT-8 (Pierce Endogen, Rockford, IL) and 6E10 (Acris Antibodies, Hiddenhausen, Germany) first underwent buffer exchange to PBS pH 7.0. Since antibody concentrations after buffer exchange were unknown, concentrations of Alexa-488-O-succinimidylester were determined empirically. Unbound dye was separated using a PD10 desalting column. Fluorescently labeled proteins are further referred to as tau^488^, tau^647^, and α-syn^647^.

### Phosphorylation of protein tau

Tau phosphorylation was conducted as described previously [17]. Fluorescently labeled protein tau was incubated with Glycogen Synthase Kinase 3β (Sigma, Saint Louis, MO) in a ratio of 0,018 U GSK-3 β / pmol tau in buffer containing 33 mM tris pH 7.5, 40 mM hepes pH 7.64, 100 mM NaCl, 5 mM EGTA, 3 mM MgCl_2_, 2 mM ATP, 180 mM sucrose, 0.13 mM PMSF, 0.67 mM benzamidine, 0.067% mercaptoethanol and 0.02% Brij-35. Samples were incubated for 20 h at 30 °C under constant shaking. As control, fluorescently labeled protein tau was incubated in buffer containing all components except for the enzyme, and is further referred to as mock phosphorylated tau (mTau). Protein phosphorylation was confirmed by sodium dodecyl sulfate polyacrylamide gel electrophoresis (SDS-PAGE) and western blot using antibody T46 as described previously [60]. Phosphorylated protein tau (pTau) featured a typical band shift compared to mTau, as described previously [17] (Figure 1B).

### Aggregation assay

Stock solutions of tau^488^, tau^647^, and α-syn^647^ were prepared for a final concentration of 10 molecules per focal volume, equivalent to a concentration of 10 nM to 20 nM. For coaggregation experiments, a 1:1 ratio of tau^488^ and α-syn^647^ was used. Prior to each experiment, tau samples were centrifuged at 100.000 g for 30 min to remove pre-formed aggregates, and all protein stock solutions were then controlled for pre-existing aggregates by SIFT [49]. Only samples free of pre-formed aggregates were used. All experiments were conducted in 96 well plates with a cover slide bottom, in a total sample volume of 20 μl per well. Plates were covered with adhesive film to obviate evaporation. For aggregation and coaggregation experiments, protein stock solutions were added to wells containing 50 mM tris buffer pH 7.0, 10 μM AlCl_3_, 10 μM FeCl_3_ or 1% dimethyl sulfoxide (DMSO), and measurements were started immediately. For coaggregation of tau^488^ oligomers with α-syn^647^ monomers, samples of tau^488^ were prepared as described above and pre-incubated for 90 min. Subsequently, α-syn^647^ monomers were added and measurements were started immediately. To investigate oligomer stability, a 10-fold SDS stock solution for a final concentration of 0.2% was added to each well after each experiment, and measurements were repeated. Each well was measured four times, resulting in a total duration of each experiment of 60 to 120 minutes, depending on experimental layout.

### Confocal single particle analysis

FCS and SIFT were performed on an InsightReader (Evotec-Technologies, Hamburg, Germany) with dual color excitation at 488 and 633 nm as described previously [48,49,61]. Data was collected separately for each excitation wavelength by two single-photon detectors. Analysis was performed using FCSPP evaluation software version 2.0 (Evotec-Technologies), allowing auto-correlation, cross-correlation and fluorescence intensity distribution (FIDA) analysis, and SIFT-2D evaluation software (Evotec-Technologies). For SIFT-2D analysis, photons were added up in time intervals (bins) of 40 μs and illustrated in a 2D scatterplot. Scatterplots depicted in this paper contain all photons of all consecutive measurements of one sample. Photon-weighted SIFT aggregation and coaggregation analysis was performed as described previously [49]. As cross-correlation detects small oligomeric protein aggregates with higher sensitivity, only cross-correlation data is depicted for experiments on tau aggregation and coaggregation with α-syn (for SIFT data see Additional files 1, 4, and 7).

### Mass spectroscopy

Mass spectroscopy was performed according to established protocols to verify the purity of tau protein stock solution [61].

### Statistical analysis

Normal distribution of data was determined by Shapiro-Wilk test. If normal distribution was confirmed, a two-sided student’s *t*-test preceded by Levene’s test for equality of variance was performed. A paired student’s *t*-test was used for experiments on SDS-resistance of oligomers. If normal distribution was not confirmed, Mann–Whitney *U*-test was performed. Bonferroni-adjustment for multiple testing was done where appropriate. Data is demonstrated as average of all independent samples, and a paired student’s *t*-test was performed including all independent samples. Error bars in figures show the standard error of the mean.

## Abbreviations

AD: Alzheimer’s disease; α-syn: α-synuclein; CBD: Corticobasal degeneration; DLB: Dementia with Lewy bodies; DMSO: dimethyl sulfoxide; FCS: Fluorescence correlation spectroscopy; FIDA: fluorescence intensity distribution analysis; GSK-3β: Glycogen synthase kinase 3β; mTau: Mock phosphorylated tau; NFT: Neurofibrillary tangles; PAGE: Polyacrylamide gel electrophoresis; PHF: Paired helical filaments; pTau: Phosphorylated tau; PSP: Progressive supranuclear palsy; SDS: Sodium dodecyl sulfate; SIFT: Scanning for intensely fluorescent targets.

## Competing interests

The authors declare that they have no competing interests.

## Authors’ contributions

GN^1. A, B, C; 2. A, B, C; 3. A, B^, BB^1. A, B: 2. A, C; 3. B^, JL^1. A, B; 2. C; 3. B^, JH^2. A, B, C; 3. A, B^, HK^1. B; 3. B^, AG^1. A, B; 2. A, C; 3. B^. Author contributions are abbreviated as follows: 1 = Research project: A. Conception, B. Organization, C. Execution. 2 = Statistical Analysis: A. Design, B. Execution, C. Review and Critique. 3 = Manuscript Preparation: A. Writing of the first draft, B. Review and Critique. All authors read and approved the final manuscript.

## Supplementary Material

Additional file 1 **Comparison of aggregation levels of pTau and mTau.** Comparison of aggregation levels of phosphorylated (pTau) and mock phosphorylated (mTau) protein tau in presence of different aggregation inducers. SIFT data is presented as ratios (colum / row). Measurements were taken from 15 independent samples, each sample was measured four times. Click here for file

Additional file 2 **Comparison of aggregation levels of pTau and mTau.** Comparison of aggregation levels of phosphorylated (pTau) and mock phosphorylated (mTau) protein tau in presence of different aggregation inducers. Cross-correlation data is presented as ratios (colum / row). Measurements were taken from 15 independent samples, each sample was measured four times. Click here for file

Additional file 3 **Kinetics of pTau and mTau aggregation and coaggregation.** Kinetics of pTau and mTau aggregation (tau/tau, 15 independent samples) and coaggregation of tau monomers (tau/α-syn, 16 independent samples) and oligomers (tau_oligo_/α-syn, 20 independent samples) with monomeric α-synuclein in presence of 1% DMSO, 10 μM Al^3+^, 10 μM Fe^3+^ and combinations of metal ions and DMSO. A control measurement depicting the aggregation status in the absence of inducers was defined as time point “0". While DMSO induces slow continuous tau aggregation, the protein’s coaggregation with α-syn reaches an early steady state. Al^3+^ promotes rapid initial aggregation and coaggregation only for pTau, while mTau coaggregation proceeds distinctly slower. Fe^3+^ induced coaggregation is continuous, with pTau proceeding faster than mTau. Upon combining metal ions and DMSO, both pTau and mTau show rapid aggregation and coaggregation exceeding the single inducers. Levels of significance are displayed as * = p < 0.05; ** = p <0.01; ‡ = p < 0.001. Click here for file

Additional file 4 **Comparison of coaggregation levels of pTau and mTau with α-syn.** Comparison of coaggregation levels of phosphorylated (pTau) and mock phosphorylated (mTau) protein tau with α-synuclein in presence of different aggregation inducers. SIFT data is presented as ratios (colum / row). Measurements were taken from 16 independent samples, each sample was measured four times. Click here for file

Additional file 5 **Comparison of coaggregation levels of pTau and mTau with α-syn.** Comparison of coaggregation levels of phosphorylated (pTau) and mock phosphorylated (mTau) protein tau with α-synuclein in presence of different aggregation inducers. Cross-correlation data is presented as ratios (colum / row). Measurements were taken from 16 independent samples, each sample was measured four times. Click here for file

Additional file 6 **Quantification of mixed tau and α-syn oligomer sizes.** Fluorescence intensity distribution analysis (FIDA) shows the size of metal ion induced mixed tau and α-syn oligomers before and after addition of SDS. SDS stable mixed oligomers can further be demonstrated in SIFT-2D analysis. Click here for file

Additional file 7 **Separation of SDS stable mixed tau and α-syn oligomers by gel filtration.** Mixed tau and α-syn oligomers induced by Al^3+^ are separated from monomers applying gel filtration. SDS-stable oligomers are eluted before tau and α-syn monomers, as demonstrated by cross-correlation and SIFT analysis. Click here for file

Additional file 8 **Comparison of coaggregation levels of pTau and mTau oligomers with α-syn.** Comparison of coaggregation levels of phosphorylated (pTau) and mock phosphorylated (mTau) tau oligomers with monomeric α-synuclein in presence of different aggregation inducers. SIFT data is presented as ratios (colum / row). Measurements were taken from 20 independent samples, each sample was measured four times. Click here for file

Additional file 9 **Comparison of coaggregation levels of pTau and mTau oligomers with α-syn.** Comparison of coaggregation levels of phosphorylated (pTau) and mock phosphorylated (mTau) tau oligomers with monomeric α-synuclein in presence of different aggregation inducers. Cross-correlation data is presented as ratios (colum / row). Measurements were taken from 20 independent samples, each sample was measured four times. Click here for file
