# Supplementary material for: Synergistic influence of phosphorylation and metal ions on tau oligomer formation and coaggregation with α-synuclein at the single molecule level
Source: Mol Neurodegener. 2012 Jul 23;7:35. doi: 10.1186/1750-1326-7-35 (PMC3472288; doi:10.1186/1750-1326-7-35)
Supplement: Additional file 1 — Comparison of aggregation levels of pTau and mTau. Comparison of aggregation levels of phosphorylated (pTau) and mock phosphorylated (mTau) protein tau in presence of different aggregation inducers. SIFT data is presented as ratios (colum / row). Measurements were taken from 15 independent samples, each sample was measured four times. [file 1750-1326-7-35-S1.pdf]

Table 1

SIFT analysis of pTau and mTau aggregation levels

		pTau			
		TRIS	DMSO 1%	Al	DMSO + Al
**pTau**	TRIS	1,00	7,29	45,72	47,87
	DMSO 1%	0,14	1,00	6,27	6,56
	Al	0,02	0,16	1,00	1,05
	DMSO + Al	0,02	0,15	0,96	1,00

		pTau			
		TRIS	DMSO 1%	Al	DMSO + Al
**mTau**	TRIS	0,22	1,60	10,03	10,50
	DMSO 1%	0,03	0,24	1,51	1,58
	Al	0,02	0,17	1,07	1,12
	DMSO + Al	0,02	0,16	1,03	1,08

		mTau			
		TRIS	DMSO 1%	Al	DMSO + Al
**mTau**	TRIS	1,00	6,65	9,34	9,75
	DMSO 1%	0,15	1,00	1,40	1,47
	Al	0,11	0,71	1,00	1,04
	DMSO + Al	0,10	0,68	0,96	1,00

Table 1: Comparison of aggregation levels of phosphorylated (pTau) and mock phosphorylated (mTau) protein tau in presence of different aggregation inducers. SIFT data is presented as ratios (colum / row). Measurements were taken from 15 independent samples, each sample was measured four times.
