# Supplementary material for: Synergistic influence of phosphorylation and metal ions on tau oligomer formation and coaggregation with α-synuclein at the single molecule level
Source: Mol Neurodegener. 2012 Jul 23;7:35. doi: 10.1186/1750-1326-7-35 (PMC3472288; doi:10.1186/1750-1326-7-35)
Supplement: Additional file 2 — Comparison of aggregation levels of pTau and mTau. Comparison of aggregation levels of phosphorylated (pTau) and mock phosphorylated (mTau) protein tau in presence of different aggregation inducers. Cross-correlation data is presented as ratios (colum / row). Measurements were taken from 15 independent samples, each sample was measured four times. [file 1750-1326-7-35-S2.pdf]

Table 2

Cross-correlation analysis of pTau and mTau aggregation levels

		pTau			
		TRIS	DMSO 1%	Al	DMSO + Al
**pTau**	TRIS	1,000	2,542	355,6	378,8
	DMSO 1%	0,393	1,000	139,9	149,0
	Al	0,003	0,007	1,000	1,065
	DMSO + Al	0,003	0,007	0,939	1,000

		pTau			
		TRIS	DMSO 1%	Al	DMSO + Al
**mTau**	TRIS	0,234	0,595	83,19	88,63
	DMSO 1%	0,065	0,166	23,29	24,81
	Al	0,004	0,011	1,484	1,580
	DMSO + Al	0,004	0,009	1,253	1,334

		mTau			
		TRIS	DMSO 1%	Al	DMSO + Al
**mTau**	TRIS	1,000	3,572	56,08	66,42
	DMSO 10%	0,188	0,673	10,53	12,51
	DMSO 1%	0,280	1,000	15,70	18,59
	Al	0,018	0,064	1,000	1,184
	DMSO + Al	0,015	0,054	0,844	1,000

Table 2: Comparison of aggregation levels of phosphorylated (pTau) and mock phosphorylated (mTau) protein tau in presence of different aggregation inducers. Cross-correlation data is presented as ratios (colum / row). Measurements were taken from 15 independent samples, each sample was measured four times.
