# Supplementary material for: Synergistic influence of phosphorylation and metal ions on tau oligomer formation and coaggregation with α-synuclein at the single molecule level
Source: Mol Neurodegener. 2012 Jul 23;7:35. doi: 10.1186/1750-1326-7-35 (PMC3472288; doi:10.1186/1750-1326-7-35)
Supplement: Additional file 3 — Kinetics of pTau and mTau aggregation and coaggregation. Kinetics of pTau and mTau aggregation (tau/tau, 15 independent samples) and coaggregation of tau monomers (tau/α-syn, 16 independent samples) and oligomers (tauoligo/α-syn, 20 independent samples) with monomeric α-synuclein in presence of 1% DMSO, 10 μM Al3+, 10 μM Fe3+ and combinations of metal ions and DMSO. A control measurement depicting the aggregation status in the absence of inducers was defined as time point “0". While DMSO induces slow continuous tau aggregation, the protein’s coaggregation with α-syn reaches an early steady state. Al3+ promotes rapid initial aggregation and coaggregation only for pTau, while mTau coaggregation proceeds distinctly slower. Fe3+ induced coaggregation is continuous, with pTau proceeding faster than mTau. Upon combining metal ions and DMSO, both pTau and mTau show rapid aggregation and coaggregation exceeding the single inducers. Levels of significance are displayed as * = p < 0.05; ** = p <0.01; ‡ = p < 0.001. [file 1750-1326-7-35-S3.pdf]

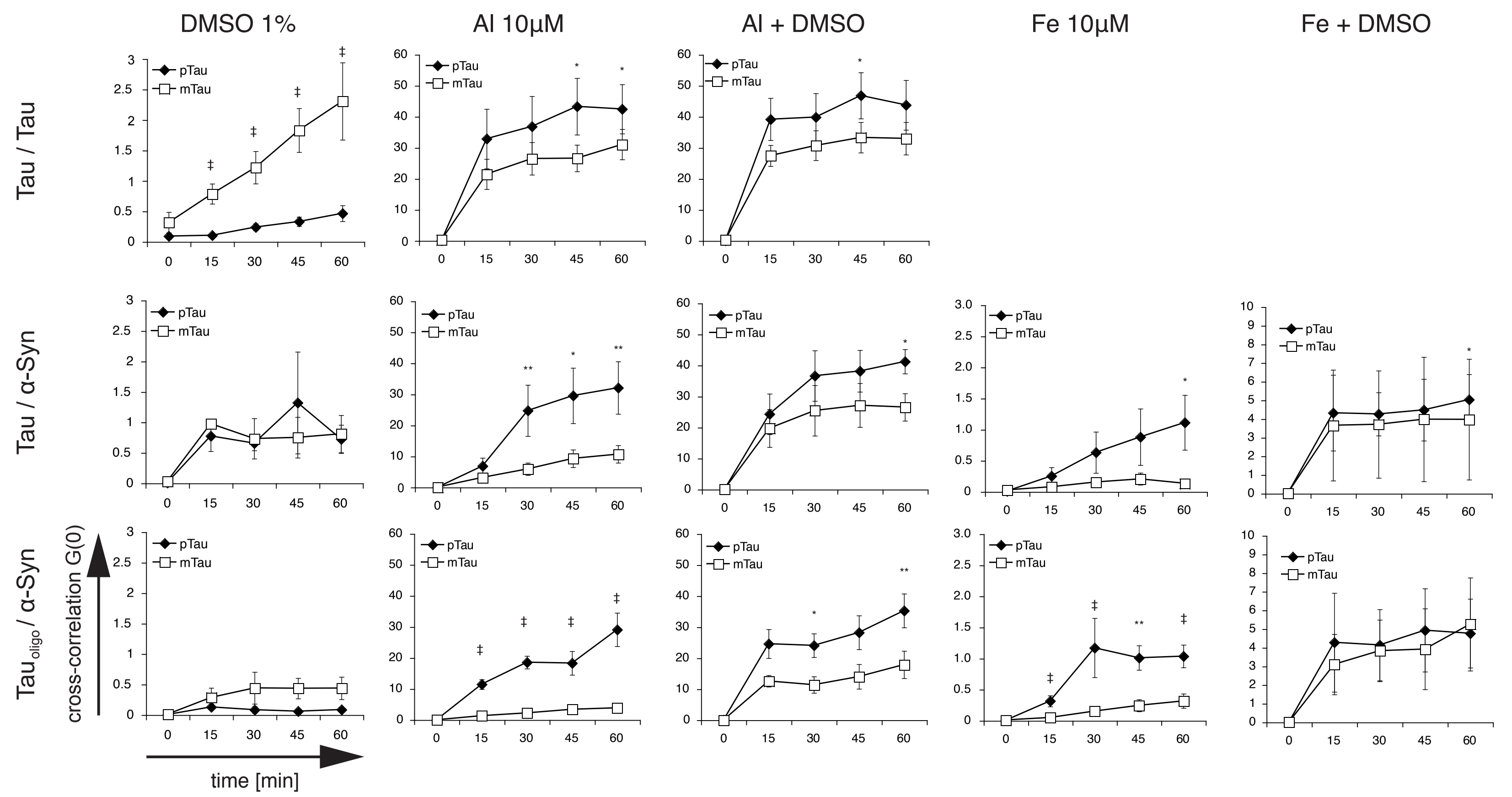

Figure 1: Kinetics of pTau and mTau aggregation (tau/tau, 15 independent samples) and coaggregation of tau monomers (tau/α-syn, 16 independent samples) and oligomers (tau$_{oligo}$/α-syn, 20 independent samples) with monomeric α-synuclein in presence of 1 % DMSO, 10 μM Al^{3+}, 10 μM Fe^{3+} and combinations of metal ions and DMSO. A control measurement depicting the aggregation status in the absence of inducers was defined as time point "0". While DMSO induces slow continuous tau aggregation, the protein's coaggregation with α-syn reaches an early steady state. Al^{3+} promotes rapid initial aggregation and coaggregation only for pTau, while mTau coaggregation proceeds distinctly slower. Fe^{3+} induced coaggregation is continuous, with pTau proceeding faster than mTau. Upon combining metal ions and DMSO, both pTau and mTau show rapid aggregation and coaggregation exceeding the single inducers. Levels of significance are displayed as * = $p < 0.05$; ** = $p < 0.01$; ‡ = $p < 0.001$
