# Supplementary material for: Synergistic influence of phosphorylation and metal ions on tau oligomer formation and coaggregation with α-synuclein at the single molecule level
Source: Mol Neurodegener. 2012 Jul 23;7:35. doi: 10.1186/1750-1326-7-35 (PMC3472288; doi:10.1186/1750-1326-7-35)
Supplement: Additional file 4 — Comparison of coaggregation levels of pTau and mTau with α-syn. Comparison of coaggregation levels of phosphorylated (pTau) and mock phosphorylated (mTau) protein tau with α-synuclein in presence of different aggregation inducers. SIFT data is presented as ratios (colum / row). Measurements were taken from 16 independent samples, each sample was measured four times. [file 1750-1326-7-35-S4.pdf]

Table 3

SIFT analysis of pTau and mTau coaggregation levels with α-syn

		pTau					
		TRIS	DMSO 1%	Fe	Al	Fe+DMSO	Al+DMSO
**pTau**	TRIS	1,000	403,6	146,4	1818,1	565,7	1784,5
	DMSO 1%	0,002	1,000	0,363	4,505	1,402	4,421
	Fe 10μM	0,007	2,76	1,000	12,42	3,863	12,19
	Al 10μM	0,001	0,222	0,081	1,000	0,311	0,982
	Fe 10μM + DMSO 1%	0,002	0,714	0,259	3,214	1,000	3,155
	Al 10μM + DMSO 1%	0,001	0,226	0,082	1,019	0,317	1,000

		pTau					
		TRIS	DMSO 1%	Fe	Al	Fe+DMSO	Al+DMSO
**mTau**	TRIS	0,668	269,5	97,78	1214,2	377,8	1191,7
	DMSO 1%	0,002	0,834	0,303	3,758	1,169	3,689
	Fe 10μM	0,026	10,64	3,860	47,94	14,92	47,05
	Al 10μM	0,001	0,384	0,139	1,728	0,538	1,696
	Fe 10μM + DMSO 1%	0,002	0,945	0,343	4,256	1,324	4,177
	Al 10μM + DMSO 1%	0,001	0,268	0,097	1,207	0,375	1,184

		mTau					
		TRIS	DMSO 1%	Fe	Al	Fe+DMSO	Al+DMSO
**mTau**	TRIS	1,000	323,1	25,33	702,5	285,3	1006,2
	DMSO 1%	0,003	1,000	0,078	2,174	0,883	3,114
	Fe 10μM	0,039	12,76	1,000	27,74	11,26	39,73
	Al 10μM	0,001	0,460	0,036	1,000	0,406	1,432
	Fe 10μM + DMSO 1%	0,004	1,133	0,089	2,463	1,000	3,527
	Al 10μM + DMSO 1%	0,001	0,321	0,025	0,698	0,284	1,000

Table 3: Comparison of coaggregation levels of phosphorylated (pTau) and mock phosphorylated (mTau) protein tau with α-synuclein in presence of different aggregation inducers. SIFT data is presented as ratios (colum / row). Measurements were taken from 16 independent samples, each sample was measured four times.
