# Supplementary material for: Synergistic influence of phosphorylation and metal ions on tau oligomer formation and coaggregation with α-synuclein at the single molecule level
Source: Mol Neurodegener. 2012 Jul 23;7:35. doi: 10.1186/1750-1326-7-35 (PMC3472288; doi:10.1186/1750-1326-7-35)
Supplement: Additional file 5 — Comparison of coaggregation levels of pTau and mTau with α-syn. Comparison of coaggregation levels of phosphorylated (pTau) and mock phosphorylated (mTau) protein tau with α-synuclein in presence of different aggregation inducers. Cross-correlation data is presented as ratios (colum / row). Measurements were taken from 16 independent samples, each sample was measured four times. [file 1750-1326-7-35-S5.pdf]

Table 4

Cross-correlation analysis of pTau and mTau coaggregation levels with α-syn

		pTau					
		TRIS	DMSO 1%	Fe	Al	Fe+DMSO	Al+DMSO
**pTau**	TRIS	1,000	36,16	30,54	1158,2	225,3	1821,7
	DMSO 1%	0,028	1,000	0,845	32,04	6,233	50,39
	Fe 10μM	0,033	1,184	1,000	37,92	7,378	59,65
	Al 10μM	0,001	0,031	0,026	1,000	0,195	1,573
	Fe 10μM + DMSO 1%	0,004	0,160	0,136	5,140	1,000	8,085
	Al 10μM + DMSO 1%	0,001	0,020	0,017	0,636	0,124	1,000

		pTau					
		TRIS	DMSO 1%	Fe	Al	Fe+DMSO	Al+DMSO
**mTau**	TRIS	0,717	25,91	21,89	830,0	161,5	1305,4
	DMSO 1%	0,025	0,918	0,775	29,41	5,721	46,25
	Fe 10μM	0,208	7,508	6,343	240,5	46,80	378,3
	Al 10μM	0,003	0,117	0,099	3,748	0,729	5,895
	Fe 10μM + DMSO 1%	0,005	0,196	0,165	6,266	1,219	9,855
	Al 10μM + DMSO 1%	0,001	0,029	0,025	0,934	0,182	1,469

		mTau					
		TRIS	DMSO 1%	Fe	Al	Fe+DMSO	Al+DMSO
**mTau**	TRIS	1,000	28,23	3,451	221,5	132,5	888,4
	DMSO 1%	0,035	1,000	0,122	7,846	4,693	31,48
	Fe 10μM	0,290	8,180	1,000	64,18	38,39	257,5
	Al 10μM	0,005	0,127	0,016	1,000	0,598	4,012
	Fe 10μM + DMSO 1%	0,008	0,213	0,026	1,672	1,000	6,707
	Al 10μM + DMSO 1%	0,001	0,032	0,004	0,249	0,149	1,000

Table 4: Comparison of coaggregation levels of phosphorylated (pTau) and mock phosphorylated (mTau) protein tau with α-synuclein in presence of different aggregation inducers. Cross-correlation data is presented as ratios (colum / row). Measurements were taken from 16 independent samples, each sample was measured four times.
