# Supplementary material for: Synergistic influence of phosphorylation and metal ions on tau oligomer formation and coaggregation with α-synuclein at the single molecule level
Source: Mol Neurodegener. 2012 Jul 23;7:35. doi: 10.1186/1750-1326-7-35 (PMC3472288; doi:10.1186/1750-1326-7-35)
Supplement: Additional file 6 — Quantification of mixed tau and α-syn oligomer sizes. Fluorescence intensity distribution analysis (FIDA) shows the size of metal ion induced mixed tau and α-syn oligomers before and after addition of SDS. SDS stable mixed oligomers can further be demonstrated in SIFT-2D analysis. [file 1750-1326-7-35-S6.pdf]

A.

tau monomer + α-syn monomer

		T_0 (before SDS)		T_{100} (after SDS)	
		tau^{488}	$α\text{-}syn^{647}$	tau^{488}	$α\text{-}syn^{647}$
**control**	mTau	-	-	-	-
	pTau	-	-	-	-
**DMSO**	mTau	15	12	-	-
	pTau	11	8	-	-
**Fe^{3+}**	mTau	39	3	54	25
	pTau	53	10	77	59
**Al^{3+}**	mTau	137	80	92	82
	pTau	131	115	98	113
**Fe^{3+} + DMSO**	mTau	45	29	81	56
	pTau	67	44	94	94
**Al^{3+} + DMSO**	mTau	132	88	94	120
	pTau	127	104	110	106

B.

tau oligomer + α-syn monomer

		T_0 (before SDS)		T_{100} (after SDS)	
		tau^{488}	$α\text{-}syn^{647}$	tau^{488}	$α\text{-}syn^{647}$
**control**	mTau	-	-	-	-
	pTau	-	-	-	-
**DMSO**	mTau	27	5	-	-
	pTau	20	2	-	-
**Fe^{3+}**	mTau	175	3	81	3
	pTau	197	14	87	17
**Al^{3+}**	mTau	154	35	92	37
	pTau	151	73	88	116
**Fe^{3+} + DMSO**	mTau	206	29	111	47
	pTau	187	33	110	54
**Al^{3+} + DMSO**	mTau	155	65	85	64
	pTau	151	83	85	109

C.

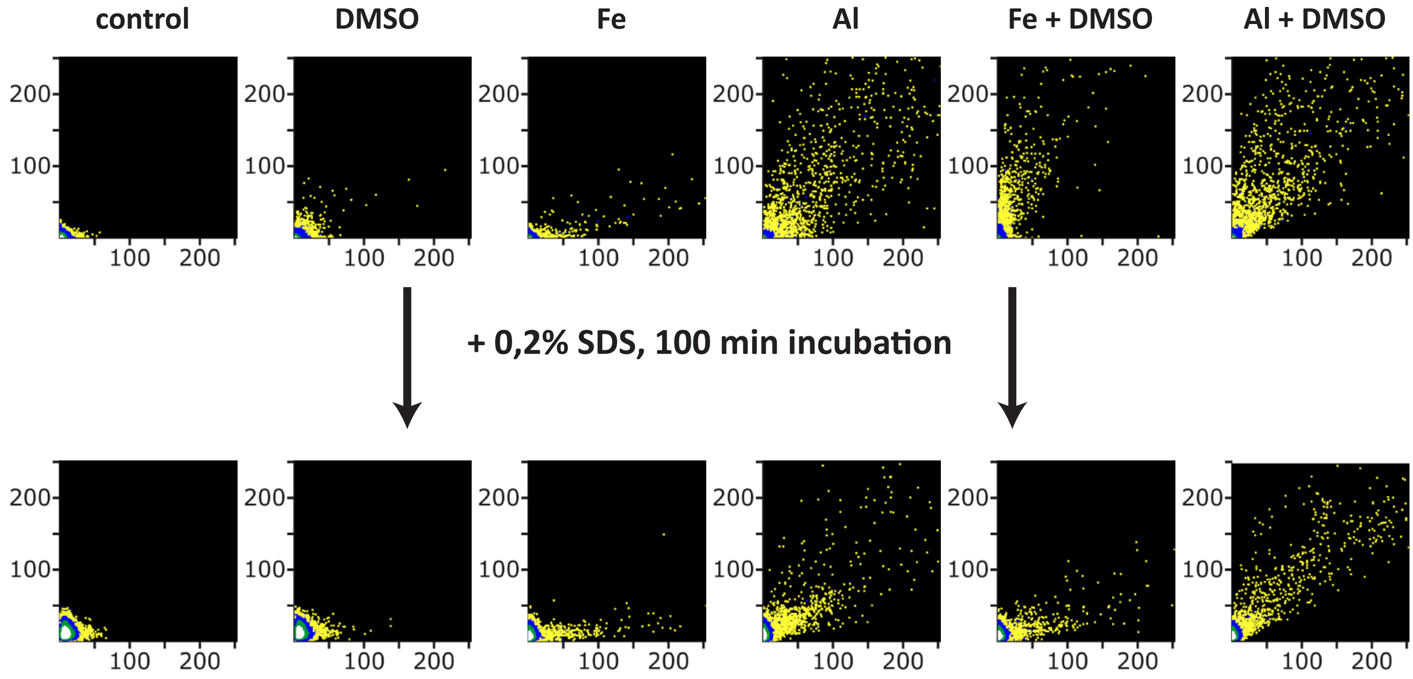

supp. figure 2: FIDA (fluorescence intensity distribution analysis) of SDS-stable mixed tau^{488} and $α\text{-}syn^{647}$ oligomers. A. Coaggregation of tau and α-syn in presence of different aggregation inducers. Oligomer size is presented as monomers per oligomer for tau^{488} and $α\text{-}syn^{647}$ before (T_0) and after (T_{100}) incubation with 0.2% SDS for 100 minutes. Oligomer size was calculated by FIDA analysis (assuming no quenching-effects in oligomers) if the presence of oligomers was indicated by cross-correlation data. B. Coaggregation of pre-formed tau^{488} oligomers and $α\text{-}syn^{647}$ monomers. Oligomer size is presented as monomers per oligomer for tau and $α\text{-}syn^{647}$ C. SIFT-2D histograms depicting the presence of mixed $pTau^{488}$ and $α\text{-}syn^{647}$ oligomers before and after incubation with 0.2% SDS for 100 minutes.
