# Supplementary material for: Synergistic influence of phosphorylation and metal ions on tau oligomer formation and coaggregation with α-synuclein at the single molecule level
Source: Mol Neurodegener. 2012 Jul 23;7:35. doi: 10.1186/1750-1326-7-35 (PMC3472288; doi:10.1186/1750-1326-7-35)
Supplement: Additional file 7 — Separation of SDS stable mixed tau and α-syn oligomers by gel filtration. Mixed tau and α-syn oligomers induced by Al3+ are separated from monomers applying gel filtration. SDS-stable oligomers are eluted before tau and α-syn monomers, as demonstrated by cross-correlation and SIFT analysis. [file 1750-1326-7-35-S7.pdf]

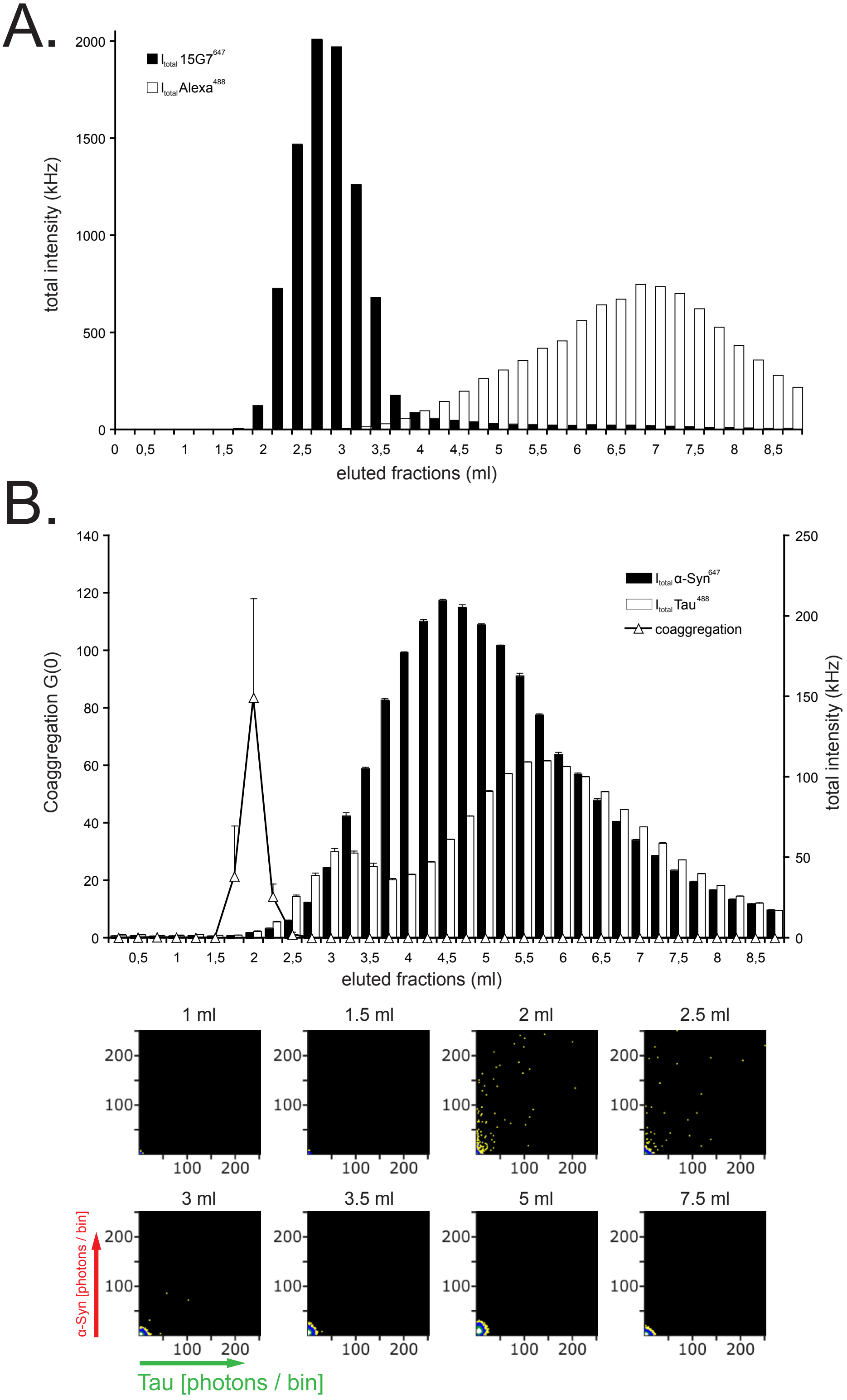

supp. figure 3: Mixed tau and α-Syn coaggregates can be separated from protein monomers by gel filtration. A. Gel filtration of fluorescently labeled IgG antibody $15G7^{647}$ (~160 kDa) and $Alexa^{488}$ fluorescent dye (0.64 kDa). A mixture containing antibody and dye was loaded onto a PD-10 gel filtration column (Invitrogen) and eluted in fractions of 0.25 ml. Fractions were then measured by SIFT. Proteins and dye are eluted in descending order according to their molecular weight. B. Tau^{488} and $\alpha\text{-Syn}^{647}$ were coincubated in presence of 100 µM Al^{3+} for one hour at room temperature and then loaded onto a PD-10 column. The eluate was collected in fractions of 0.25 ml in sample tubes containing 5 µl of a 10% SDS solution (final concentration 0.1%) to prevent protein adhesion to the sample tube. Cross-correlation amplitudes and SIFT-2D histograms show the presence of SDS-stable mixed oligomers that elute as a well-defined peak before the elution of monomers, as indicated by increases in total fluorescence intensity.
