# Supplementary material for: Synergistic influence of phosphorylation and metal ions on tau oligomer formation and coaggregation with α-synuclein at the single molecule level
Source: Mol Neurodegener. 2012 Jul 23;7:35. doi: 10.1186/1750-1326-7-35 (PMC3472288; doi:10.1186/1750-1326-7-35)
Supplement: Additional file 8 — Comparison of coaggregation levels of pTau and mTau oligomers with α-syn. Comparison of coaggregation levels of phosphorylated (pTau) and mock phosphorylated (mTau) tau oligomers with monomeric α-synuclein in presence of different aggregation inducers. SIFT data is presented as ratios (colum / row). Measurements were taken from 20 independent samples, each sample was measured four times. [file 1750-1326-7-35-S8.pdf]

Table 5

SIFT analysis of coaggregation levels of pTau and mTau oligomers with monomeric α-syn

		pTau					
		TRIS	DMSO 1%	Fe	Al	Fe+DMSO	Al+DMSO
**pTau**	TRIS	1,0000	37,03	338,4	2152,1	745,9	2777,5
	DMSO 1%	0,0270	1,0000	9,137	58,13	20,15	75,02
	Fe 10μM	0,0030	0,1094	1,0000	6,360	2,204	8,209
	Al 10μM	0,0005	0,0172	0,1572	1,0000	0,3466	1,291
	Fe 10μM + DMSO 1%	0,0013	0,0496	0,4536	2,885	1,0000	3,724
	Al 10μM + DMSO 1%	0,0004	0,0133	0,1218	0,7748	0,2685	1,0000

		pTau					
		TRIS	DMSO 1%	Fe	Al	Fe+DMSO	Al+DMSO
**mTau**	TRIS	0,5103	18,89	172,7	1098,2	380,6	1417,3
	DMSO 1%	0,0027	0,0998	0,9119	5,800	2,010	7,486
	Fe 10μM	0,0118	0,4374	3,997	25,42	8,812	32,81
	Al 10μM	0,0097	0,3610	3,299	20,98	7,272	27,08
	Fe 10μM + DMSO 1%	0,0015	0,0562	0,5136	3,267	1,132	4,216
	Al 10μM + DMSO 1%	0,0006	0,0240	0,2190	1,393	0,4827	1,797

		mTau					
		TRIS	DMSO 1%	Fe	Al	Fe+DMSO	Al+DMSO
**mTau**	TRIS	1,0000	189,3	43,19	52,34	336,1	788,6
	DMSO 1%	0,0053	1,0000	0,2281	0,2764	1,775	4,165
	Fe 10μM	0,0232	4,383	1,0000	1,212	7,782	18,26
	Al 10μM	0,0191	3,618	0,8253	1,0000	6,423	15,07
	Fe 10μM + DMSO 1%	0,0030	0,5632	0,1285	0,1557	1,0000	2,346
	Al 10μM + DMSO 1%	0,0013	0,2401	0,0548	0,0664	0,4263	1,0000

Table 5: Comparison of coaggregation levels of phosphorylated (pTau) and mock phosphorylated (mTau) tau oligomers with monomeric α-synuclein in presence of different aggregation inducers. SIFT data is presented as ratios (colum / row). Measurements were taken from 20 independent samples, each sample was measured four times.
