# Supplementary material for: Synergistic influence of phosphorylation and metal ions on tau oligomer formation and coaggregation with α-synuclein at the single molecule level
Source: Mol Neurodegener. 2012 Jul 23;7:35. doi: 10.1186/1750-1326-7-35 (PMC3472288; doi:10.1186/1750-1326-7-35)
Supplement: Additional file 9 — Comparison of coaggregation levels of pTau and mTau oligomers with α-syn. Comparison of coaggregation levels of phosphorylated (pTau) and mock phosphorylated (mTau) tau oligomers with monomeric α-synuclein in presence of different aggregation inducers. Cross-correlation data is presented as ratios (colum / row). Measurements were taken from 20 independent samples, each sample was measured four times. [file 1750-1326-7-35-S9.pdf]

Table 6

Cross-correlation analysis of coaggregation levels of pTau and mTau oligomers with monomeric α-syn

		pTau					
		TRIS	DMSO 1%	Fe	Al	Fe+DMSO	Al+DMSO
**pTau**	TRIS	1,0000	7,208	93,22	1702,1	454,7	2813,8
	DMSO 1%	0,1387	1,0000	12,93	236,2	63,09	390,4
	Fe 10μM	0,0107	0,0773	1,0000	18,26	4,88	30,19
	Al 10μM	0,0006	0,0042	0,0548	1,0000	0,2672	1,653
	Fe 10μM + DMSO 1%	0,0022	0,0159	0,2050	3,743	1,0000	6,188
	Al 10μM + DMSO 1%	0,0004	0,0026	0,0331	0,6049	0,1616	1,0000

		pTau					
		TRIS	DMSO 1%	Fe	Al	Fe+DMSO	Al+DMSO
**mTau**	TRIS	0,6850	4,938	63,86	1166,0	311,5	1927,6
	DMSO 1%	0,0273	0,1964	2,541	46,39	12,39	76,69
	Fe 10μM	0,0934	0,6735	8,710	159,1	42,49	262,9
	Al 10μM	0,0044	0,0319	0,4125	7,532	2,012	12,45
	Fe 10μM + DMSO 1%	0,0026	0,0184	0,2381	4,347	1,161	7,186
	Al 10μM + DMSO 1%	0,0007	0,0050	0,0647	1,182	0,3158	1,954

		mTau					
		TRIS	DMSO 1%	Fe	Al	Fe+DMSO	Al+DMSO
**mTau**	TRIS	1,0000	25,14	7,331	154,8	268,2	986,6
	DMSO 1%	0,0398	1,0000	0,2917	6,159	10,67	39,25
	Fe 10μM	0,1364	3,429	1,0000	21,12	36,59	134,6
	Al 10μM	0,0065	0,1624	0,0474	1,0000	1,733	6,373
	Fe 10μM + DMSO 1%	0,0037	0,0937	0,0273	0,5771	1,0000	3,678
	Al 10μM + DMSO 1%	0,0010	0,0255	0,0074	0,1569	0,2719	1,0000

Table 6: Comparison of coaggregation levels of phosphorylated (pTau) and mock phosphorylated (mTau) tau oligomers with monomeric α-synuclein in presence of different aggregation inducers. Cross-correlation data is presented as ratios (colum / row). Measurements were taken from 20 independent samples, each sample was measured four times.
